# Prevalence of Traumatic Dental Injuries and Associated Risk Factors Among Preschool Children in Goa: A Cross-Sectional Study

**DOI:** 10.7759/cureus.73894

**Published:** 2024-11-18

**Authors:** Richa R Borkar, Elaine S Barretto, Dinesh F Swamy, Elaine V Fijardo

**Affiliations:** 1 Department of Pediatric and Preventive Dentistry, Goa Dental College and Hospital, Bambolim, IND

**Keywords:** cross-sectional studies, humans, pediatric dentistry, preschool children, prevalence, risk factors, tooth injuries epidemiology, traumatology

## Abstract

Background and aim: Primary teeth trauma has received very little attention compared to permanent teeth due to their eventual exfoliation. Enamel discolorations, hypoplasias, and enamel-surface abnormalities have been reported in the permanent dentition following primary tooth trauma. Traumatic dental injuries (TDI) also have an impact on the quality of life of an individual. The study aims to determine the prevalence of TDI and its association with various risk factors in preschool children in the state of Goa. The primary objective is to determine the prevalence of TDI in primary anterior teeth in preschool children in the state of Goa. The secondary objective is to determine the association of various risk factors with TDI in preschool children in the state of Goa.

Methods: A cross-sectional survey was conducted among preschool children aged three to five years across the state of Goa. A total of 971 children were included in the study. Parents or guardians of participants who reported trauma answered an interview addressing the history of the injury. The anterior dental trauma and associated factors such as age, gender, time of injury, month of injury, place of injury, occlusal relationship, lip competence, oral habits, and ordinal position of the child were assessed and analyzed.

Results: Out of the 971 children examined, TDI was reported in 157 (16.1%) children. Of these, 83 of 157 (52.8%) children had experienced TDI at home. Three-year-old children had the highest TDI prevalence of any age group, with 32 of 145 (22.0%) experiencing TDI. The most commonly affected tooth (100 of 233, 10.3%) was the primary maxillary right central incisor. Of the 233 traumatized teeth, 100 (42.9%) had code 2 injuries (enamel fractures only), which were the most frequently reported injuries. Mesio-incisal angle fracture was detected in 84 (34.3%) teeth affected with TDI. With respect to the number of teeth traumatized, 99 of 157 (63.0%) of children had a single tooth traumatized. Among all children who had sustained TDI, 103 (65.6%) were firstborns, 50 (31.8%) were secondborns, and four (2.5%) were thirdborn children. Only eight children received treatment, of which seven received medications and only one child received dental treatment. Parents being unaware of TDI was the main reason for not seeking treatment.

Conclusions: This study highlights the critical need for educating parents and teachers about TDI prevention and the importance of prompt dental care. The findings offer valuable insights for formulating state- and national-level policies and preventive strategies for managing TDI.

## Introduction

Traumatic dental injuries (TDI) have been reported as the fifth most prevalent disease or injury after caries, tension headaches, iron deficiency anemia, and age-related or other hearing loss [1]. A systematic review and meta-analysis conducted in 2018 have reported a worldwide prevalence of TDI ranging from 13.0% to 17.4% in the permanent dentition and 17.3% and 28.7% in the primary dentition, with roughly one-third of preschoolers having experienced some form of oral trauma [2].

The slow evolution from crawling to walking and thereafter the gradual maturation of locomotor abilities is accompanied by a surge in the incidence of falls, resulting in TDI to the primary dentition. Damage to the primary teeth might potentially harm the permanent dentition since the primary tooth roots lie in close proximity to the permanent tooth germs. The common consequences of primary teeth trauma to the succedaneous teeth are enamel discolorations, hypoplasias, and enamel-surface abnormalities. Apart from the obvious biological impact, dental trauma can also trigger a cascade of adverse socioeconomic effects, affecting self-esteem and quality of life, causing absence from school or work, disrupting sleep, and altering daily routines [3].

Every TDI case is made unique by the infinite combinations of environmental and patient-related factors. Due to this variation, the development of and reliance on evidence-based practices is the most efficient approach to TDI management. To this end, data collection and population-level investigation of trends and causes are of prime significance.

It is evident from the population-based studies conducted on TDIs that permanent teeth injuries have received more attention than those to primary teeth. This is probably because primary teeth are thought to exfoliate eventually and don't need as much care as permanent teeth [4]. In the literature pertaining to pediatric dentistry, the TDIs are underrepresented. A profile analysis of the top ten pediatric dentistry journals from the years 2000 to 2010 revealed that the number of articles on TDI in primary dentition comprised only 3.2% of all the publications [5]. A systematic review of TDI in India, conducted by Patnana et al., has estimated the comprehensive prevalence of TDI in primary teeth to range from 18.2% to 31.4% [6]. TDI prevalence studies have been carried out in various states of India. However, such a study has not been conducted in the state of Goa to date. Hence, the present study serves as a first step in estimating and categorizing TDI within the state.

## Materials and methods

Ethical clearance was obtained from the Institutional Ethics Committee of Goa Dental College and Hospital, Bambolim, India (approval number: GDCH/IEC/VIII-2022 (08)). Permission was obtained from the Directorate of Education. This cross-sectional study was conducted in the preschools of Goa, where children between the ages of three and five were admitted. The sample size was determined to be 880, based on the prevalence of 10.2% obtained in a similar study conducted in the state of Jaipur [3]. Formula used:

\[ n = \frac{Z^2 \cdot P \cdot (1 - P)}{d^2} \]

\[ = \frac{(1.96)^2 \cdot 0.102 \cdot (1 - 0.102)}{(0.02)^2} \]

\[ = 880 \]

where n is the sample size, Z is the statistic corresponding to the level of confidence, P is the expected prevalence, and d is the margin of error or precision. Since the sample size calculation was based on the prevalence of a pre-existing study, the effect size, alpha, and beta were not taken into consideration.

A random cluster sampling method was used. The total population of Goa was divided by the DoE into 11 talukas, which are the regional administrative units in India (with Dharbandora taluka being grouped under Sanguem taluka). Each taluka formed a cluster. Simple random sampling was used to select preschools from the list of preschools registered under the DoE, Porvorim, by lottery method. For every taluka, each preschool from the list was assigned a number, and these numbers were noted down on slips of paper. The slips were then mixed in a bowl and drawn at random. Based on the drawn numbers, the preschools were taken as samples. Thus, in this method, each preschool had an equal chance of being selected for the sample.

Permission, as well as appointments for the study, were obtained from the preschools, and consent forms were handed over to the preschool in charge on the same visit to be dispatched to the parents.

The study included all children attending preschool on the day of the study whose parents or guardians were willing to provide consent. However, children were excluded if they had missing primary anterior teeth for reasons other than trauma, had erupting permanent incisors, were uncooperative during the oral examination as determined by Frankl’s Behavior Rating Scale 1 and 2, or had special health care needs.

The examination was carried out between June 2023 and August 2023. In total, 971 children had completed and signed consent forms by their parents/guardians. Keeping in mind ethical considerations, we opted to include all consenting children in our study rather than turn away any excess children. The examination was done by a calibrated dental surgeon wearing an N95 mask and single-use nitrile gloves in natural light in the classrooms as per the WHO type III criteria (inspection) with an assistant (dental surgeon) to record the data. Parents or guardians of the children who reported trauma answered a face-to-face interview addressing the history of the injury, and the children who required treatment for TDI were issued referral cards. Each check-up, including history-taking, took approximately 10 minutes. The dental trauma was assessed by the method used by Andreasen et al. [7], consisting of the visual assessment of tooth discoloration and dislocation of teeth (Table 1). Permission was obtained from the original publishers to reproduce this table. The data was collected on the paper-based data collection form and analyzed using STATA (Version 16.0, StataCorp LLC).

**Table 1:** Visual assessment of tooth discoloration and dislocation of teeth TDI: traumatic dental injury Andreasen et al. (2012) [7]

Code	Injury	Criteria
0	No injury	No evidence of treated or untreated dental injury
1	Treated dental injury	Composite restoration, bonding of the tooth fragment, crown, denture, or bridge pontics replacing missing teeth due to TDI, restoration located in the palatal/lingual surface of the crown suggesting endodontic treatment. No evidence of decay or any other treatment provided due to TDI
2	Enamel fracture only	Loss of a small portion of the crown, including only enamel
3	Enamel/dentin fracture	Loss of a portion of the crown, including enamel and dentin without pulp exposure
4	Pulp injury	Signs and symptoms of pulp involvement due to dental injury. It includes fractures with pulp exposure, dislocation of the tooth, presence of sinus tract, and/or swelling in the labial/lingual vestibule without evidence of caries and discoloration of the crown. The examiner must check if pulp involvement was due to caries (presence of treated/untreated caries lesion)
5	Missing tooth due to trauma	Absence of the tooth due to a complete avulsion. Code 5 should be used only for teeth judged to be missing due to trauma. A positive history of trauma is needed to record missing teeth due to trauma, and the examiner must ask the participant if the avulsion was due to a harmful incident involving the front teeth/mouth or have been extracted due to caries
9	Excluded	Signs of traumatic injury cannot be assessed, i.e., the presence of appliances or all permanent incisors missing due to caries

TDIs in children may have occurred in a time frame of six months or longer, and the parents may not recall the details of TDI that their children experienced. This recall bias was anticipated, and separate codes were allotted for parents who could not recall TDI. The statistical tests used were the chi-square test to determine the association of TDI with the risk factors, and Pearson's chi-square test of association was used to measure the linear relationships between the risk factors.

## Results

Out of 971 children examined, 157 (16.1%) were observed to have an anterior dental trauma. The association of dental trauma with gender characteristics was not statistically significant (Table 2).

**Table 2:** Distribution of TDI by gender TDI: traumatic dental injury

Gender	Absence of TDI n (%)	Presence of TDI	Chi-square (p-value)
Boys	409 (83.47%)	81 (16.53%)	0.096 (0.757)
Girls	405 (83.83%)	76 (15.8%)	

**Figure 1:**
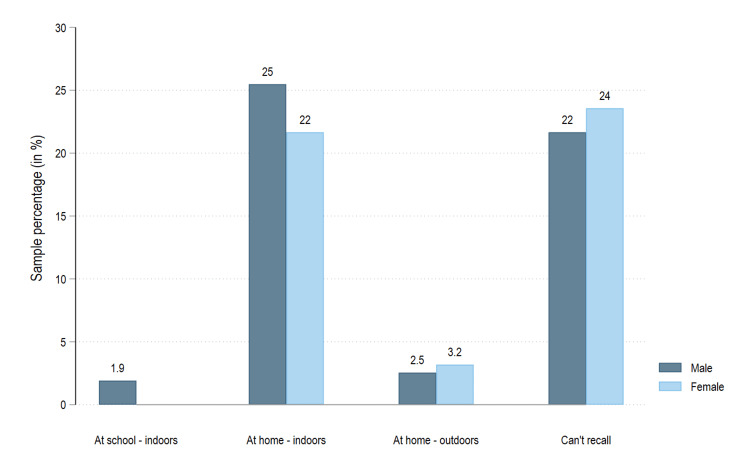
Distribution of TDI by place of injury and gender TDI: traumatic dental injury

Three-year-old children had the highest TDI prevalence of any age group, with 32 (22.0%) out of 145 experiencing TDI. An equal number of boys and girls were affected; 15 (1.6%) boys and 15 (1.6%) girls aged three experienced trauma. A slightly higher prevalence was noted among boys aged four and five. Figure 1 shows that most TDI incidents among boys and girls occurred indoors at home (47.1%).

Among the children with TDI, 74 (47.1%) parents recalled injuries that had occurred in excess of the last six months; eight (5.1%) could recall injuries occurring in the preceding three-to-six-month period; and 10 (6.3%) parents recalled an injury in the preceding one-to-three-month period.

Parents of 71 (34.3%) children affected with TDI could not recall any history of trauma occurring in their child. With 11 (7.0%) affected children, July had the highest number of TDI cases recorded, whereas only one (0.8%) TDI was reported in November.

The most commonly affected tooth was the primary maxillary right central incisor. Of the 233 traumatized teeth, 100 (42.9%) had code 2 injuries (enamel fractures only), which were the most frequently reported injuries, while 65 (27.9%), 59 (25.3%), and nine (3.9%) teeth had code 3 (enamel/dentin fracture), code 4 (pulp injury), and code 5 (missing tooth due to trauma) injuries, respectively, as illustrated in Table 3.

**Table 3:** Distribution of TDI by the type of fracture TDI: traumatic dental injury

Code	Type of fracture	N (%)
0	No injury	0
1	Treated dental injury	0
2	Enamel fracture only	100 (42.9%)
3	Enamel/dentin fracture	65 (27.9%)
4	Pulp injury	59 (25.3%)
5	Missing tooth due to trauma	9 (3.9%)
9	Excluded tooth	0
Total		233

Among the children with TDI, 99 (63.0%) had a single tooth traumatized, while 45 (28.6%) had two teeth traumatized, eight (5.1%) had three teeth traumatized, and five (3.1%) had four teeth traumatized. Table 4 represents the distribution of different types of fracture lines among the teeth affected by TDI. Mesio-incisal angle fracture was detected in 84 (34.3%) teeth affected with TDI, while 56 (24.0%) teeth exhibited disto-incisal angle fractures, 37 (15.8%) teeth had incisal-edge-only fractures, and 20 (8.5%) teeth had horizontal fractures. There was no fracture line detected in 36 (15.4%) teeth, despite evidence of trauma.

**Table 4:** Distribution of TDI by type of fracture line TDI: traumatic dental injury

Type of fracture line	N (%)
No fracture line	36 (15.7%)
Mesio incisal	80 (34.9%)
Disto incisal	56 (24.5%)
Horizontal	20 (8.7%)
Incisal edge-only	37 (16.16%)

Evidence of dental caries was seen in only 70 (30.8%) teeth with TDI, while 157 (69.2%) teeth with TDI were cavity-free. The majority of children with TDI had a mesial-step occlusal relationship, with 109 (69.4%) children revealing a right-side mesial-step occlusal relationship and 113 (71.9%) revealing a left-side mesial-step occlusal relationship. Competent lips were noted in 151 (96.1%) children with TDI, while four (2.5%) had potentially incompetent lips, and only two (1.2%) had incompetent lips; 131 (83.4%) children with TDI did not report any oral habits.

Among all children who had sustained TDI, 103 (65.6%) were firstborns, 50 (31.8%) were secondborn children, and 4 (2.5%) were thirdborn children. Table 5 depicts that only eight children received treatment, of which seven children received medications and only one child received dental treatment.

**Table 5:** Distribution of the time duration between TDI and treatment sought and nature of treatment received TDI: traumatic dental injury

Treatment sought	Treatment received		
	Pulp treatment	Medication	Other
Within 24 hours	-	3	1
After 24 hours	-	1	-
After 7 days	1	2	-

**Figure 2:**
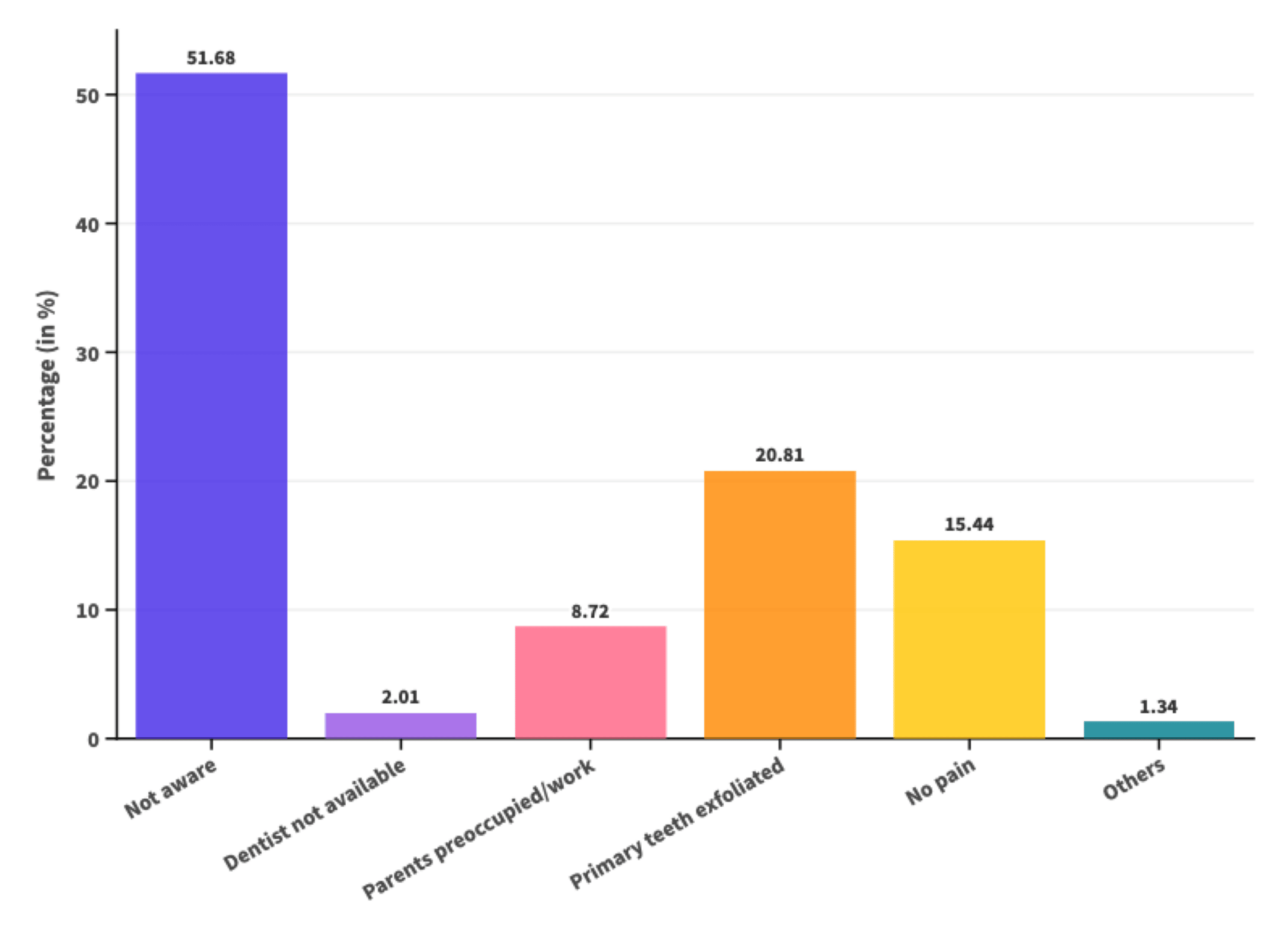
Graphical representation of reasons for not seeking treatment among parents of children who had suffered TDI TDI: traumatic dental injury

Figure 2 represents the various reasons given by the parents for not seeking treatment. The primary reason for not seeking treatment among 77 (51.68%) children's parents was their "unawareness of TDI." "Primary teeth will exfoliate" was cited by 31 (20.8%), while 23 (15.44%) did not seek treatment as "the child never complained of any pain or discomfort."

## Discussion

TDI has been reported as the fifth most prevalent disease or injury after caries, tension headaches, iron deficiency anemia, and age-related or other hearing loss [1]. A systematic review and meta-analysis conducted in 2018 have reported a worldwide prevalence of TDI ranging from 13.0% to 17.4% in the permanent dentition and 17.3% to 28.7% in the primary dentition, with roughly one-third of preschoolers having experienced some form of oral trauma [2].

In the literature pertaining to pediatric dentistry, the TDIs are underrepresented. A profile analysis of the top 10 pediatric dentistry journals from the years 2000 to 2010 revealed that the number of articles on TDI in primary dentition comprised only 3.2% of all the publications [5]. A systematic review of TDI in India, conducted by Patnana et al., has estimated the comprehensive prevalence of TDI in primary teeth to range from 18.24% to 31.43% [6]. TDI prevalence studies have been carried out in various states of India. However, such a study has not been conducted in the state of Goa to date. Hence, the present study serves as a first step in estimating and categorizing TDI within the state.

In Goa, 157 (16.16%) preschool children were examined to have TDIs. This percentage is higher than the findings of Chalissery et al. (10.2%) [3], Kiran et al. (12.2%) [8], and Shekhar et al. (6.2%) [9], but lower than the results reported by Dutra et al. (47%) [10], ElKarmi et al. (26.4%) [4], and Berti et al. (52.3%) [11].

In the current study, risk factors of TDI like age, gender, time, month, and place of injury, caries, occlusal plane relationship, lip competence, oral habits, and ordinal position of the child were taken into consideration. Another aspect of the present study was its retrospective nature. Participants in retrospective investigations may give incorrect responses to questions that depend on their memory of events that occurred months or even years earlier, leading to significant sources of bias and error. In our study as well, 74 (47.1%) TDIs occurred in a time frame of six months or later, and 54 (34.3%) parents were unable to recall the TDI that their children had previously experienced. Hence, in an effort to reduce the inherent bias that was anticipated in the present study, separate codes were allotted for parents who could not recall TDI.

The three-year-old cohort presented the highest incidence of TDI, accounting for 32 (22.0%) of 145 afflicted children. The four- and five-year-old cohorts had the lowest incidence, accounting for 59 (17%) of 347 and 66 (16.1%) of 479 affected children, respectively. This is in accordance with the findings of Assunção et al. [12] and de Amorim Lde et al. [13]. The higher vulnerability of the younger age groups can be attributed to the delay in children achieving mastery in motor skills for walking and running and to their behavioral traits that involve inquisitiveness and a lack of threat perception. However, the findings of Sharma et al. [14] and Shekhar et al. [9] found that the incidence of TDI increased as children aged from two to five years. Societal changes, such as the rise in outdoor and physical sporting activities for young children replacing previous conservative parenting that kept very young children and preschoolers indoors, might be responsible for such a changing trend.

In the current study, a nearly equal number of boys and girls (81 (16.5%) boys and 76 (15.8%) girls) had TDI across the ages of three, four, and five, with a slightly higher male prevalence, but this difference was not statistically significant. This could be attributed to the fact that children less than six years of age are exposed to the same type of risk factors, regardless of gender. However, according to Altun et al. [15], boys experienced dental hard tissue and pulp injuries more frequently than girls.

Accidental falls are one of the main reasons for TDIs. The state of Goa has the monsoon phenomenon prevalent between July and September, with peak rainfall being received during the onset and early phases of the monsoon. One possible explanation for our results showing the highest reported prevalence in July could be due to the children being indoors during heavy rainfall. Literature on the subject is varied, with certain authors reporting TDI prevalence increasing in spring, followed by winter, with peaks corresponding to March and May [16], while others have reported peaks occurring in spring [17] or summer [18]. Hence, we have refrained from comparing our results with those of other studies conducted in different geographical areas, as weather-related self-falls during the winter season do not apply to our study. Similarly, socio-cultural norms and behaviors associated with seasons vary across cultures and may explain the variation in results from research conducted in areas outside the South Asian subcontinent. In the current study, 74 (47.13%) TDIs occurred indoors at home. This is in accordance with various other studies conducted by Dutra et al. [10], Norton et al. [19], ElKarmi et al. [4], and Tewari et al. [20]. This can be explained by the fact that children under the age of six spend most of their time at home.

The primary maxillary central incisors were the most commonly affected teeth, followed in frequency by the primary maxillary lateral incisors, with 194 (19.9%) primary maxillary central incisors and 37 (3.8%) primary maxillary lateral incisors affected. This finding was consistent with the findings of Agostini et al. [21] and Al-Majed et al. [22] and is due to the fact that they are generally more proclined than the other teeth.

In our study, canines showed no involvement, and the mandibular teeth received minimal trauma. According to de Amorim Lde et al. [13], the mandible is protected by the maxilla during occlusion. Additionally, the flexible mandible tends to lessen the impact forces directed to the lower anterior teeth, whereas the upper jaw is inflexible due to its fixity to the skull.

Our research confirms the findings of Choi et al. [18] and Robson et al. [23], who report single-tooth fracture to be the most common, followed in frequency by two teeth, three teeth, and four or more teeth. The severity of TDI can range from minor enamel fractures to significant tooth displacement or avulsion. The majority of injuries reported in the current study were uncomplicated crown fractures, with enamel-only fractures accounting for 100 (42.9%) of the teeth and outnumbering enamel-dentin fractures, which were observed in 65 (27.9%) of the teeth. Enamel fractures have also been reported in the literature to be the most common type of TDI in the primary teeth [22]. According to Agostini et al., short root lengths of primary teeth along with the elastic nature of supporting tissues account for a significant percentage of luxation injuries in the primary dentition following low-intensity impacts [21]. However, luxation injuries go unnoticed during the inspection in the field due to poor sight, inadequate lighting, and a lack of suitable diagnostic tools, accounting for their low prevalence in the literature.

The current study identified four different types of fracture lines, in decreasing order of their frequency: fractures involving the mesio-incisal angle were seen in 84 (34.3%) teeth, 56 (24.0%) teeth were afflicted with disto-incisal angle fractures, 37 (15.8%) exhibited incisal edge-only, and 20 (8.5%) demonstrated horizontal fracture. The incisal edge-only fracture in the present study was more frequently observed than the horizontal fractures. To the best of our knowledge, incisal-edge fractures that do not involve the mesial and distal incisal angles have not been documented in the literature. According to the anatomy of the incisal edge of the central incisor, the distal incisal angle is thicker and more rounded than the mesial incisal angle, making it more prone to fracture under relatively lighter impact forces than the distal angle. The incidence of fracture patterns involving the mesial incisal edges is reported to be roughly two times higher than that of fracture patterns involving the distal incisal angles [24].

No significant association was found between caries and TDI. The current study found a higher TDI in non-carious teeth. This was in accordance with a study conducted by Siqueira et al. [25]. These researchers have commented that trauma to teeth is more likely to be caused by extrinsic causes than by the teeth being weakened by dental caries alone.

In the present study, most of the children who had experienced a TDI exhibited a mesial-step terminal relationship. Depending on the availability of spaces in the primary dentition and the growth pattern of the maxilla and mandible, the mesial step in the primary dentition may turn into a class I or class III molar relationship in the permanent dentition. Therefore, Shekhar et al. [9] opined that it would be unsuitable to attribute mesial-step as a possible risk factor for trauma to the incisors. Out of the 157 children who reported trauma in our study, 151 (96.1%) had competent lips, which is in contrast to Robson et al. [23], who reported inadequate lip coverage as a predisposing factor to TDI. However, in agreement with our research, Born et al. [26] and Borzabadi-Farahani et al. [27] did not report any association between lip incompetence and maxillary incisor trauma. An increased overjet has been directly related to lip incompetence, but since preschoolers generally do not receive interceptive orthodontic therapy to prevent TDI, Arraj et al. [28] recommended that caregivers be advised about the risk of TDI when an overjet ≥3 mm is noted in the primary dentition. As in the current study, Norton and O'Connell also did not report any statistically significant association between TDI and oral habits [19].

The impact of birth order on TDI has not been extensively studied. In the current study, first-born children had the highest prevalence of TDI, with second- and third-born children following in frequency. Our study is in agreement with the conclusion reached by Orton et al., who said that TDIs may occur more frequently in older siblings [29]. However, it contrasts with the findings of Siqueira et al., who found no effect of birth order on the occurrence of TDI [25].

In consonance with the findings of our study, a body of literature has confirmed that after a traumatic event, the degree of the dental injury did not affect whether the child sought dental care after the traumatic event; this included even the more serious dental injuries like avulsions and intrusions. Seventy-seven (51.6%) parents were ignorant of how crucial it is to undergo care following a TDI, and 31 (20.8%) parents did not seek treatment as the afflicted teeth "were primary teeth, which would ultimately exfoliate," while 23 (15.4%) did not seek treatment as "the child never complained of any discomfort." Garcia-Godoy et al. suggested that the parental decision to seek treatment is dictated by a preference for treating injuries that affect aesthetics (fractures or changes in tooth color following luxation injuries) or when they see their child crying or bleeding [30]. In the absence of such stimuli, very few parents were actually concerned about the possible effects of trauma on their primary and permanent teeth. This could explain why parents of children with TDI who were unaware of the TDIs or did not have an actively distressed child did not seek any treatment.

With regards to the time elapsed between injury and seeking treatment, our study findings revealed only four subjects receiving treatment within 24 hours. Overall, only eight of the 157 subjects with TDIs sought treatment in the current study. The same biases as discussed previously are likely due to the perception that primary teeth are temporary and any injury to them is not considered a disease, as speculated by Robson et al. [23].

Due to the cross-sectional design of the present study, only injuries evident on the day of examination were recorded. In the present study and other similar study designs, the injuries to the supporting structures of the teeth could be underreported, as they may heal without leaving any sign of injury. Therefore, it is possible that the prevalence figures in reality may be somewhat higher. Another possible limitation was the bias in parents related to the lack of recall of TDI, which may have occurred in the extended past.

When a child experiences a dental injury, the parents/caregivers can consult with various healthcare facilities, which include general dental practitioners, emergency medical departments, community dental clinics, specialist dental services, and pharmaceutical professionals. Based on the findings of the current study, a program can be developed to create awareness among parents to seek dental care right away if their child has a TDI. Hence, such initiatives should focus on the potential effects of trauma on the primary and permanent teeth and should be made available to school staff as well as primary care providers so they can alert parents immediately after a TDI.

## Conclusions

This study presents the first-ever assessment of TDI prevalence in the state of Goa, India, assessed to be 16.1%. It was concluded that three-year-old children had the highest prevalence of TDI. There were no statistically significant differences noted in the TDI prevalence among boys and girls. Enamel fracture was the most common type of injury noted, followed by enamel/dentin fracture, pulp injury, and avulsion. The most common type of fracture line was mesio-incisal angle fracture. There was no fracture line detected in 15.7% of teeth, despite evidence of trauma. Very few children (8 out of 157 children) sought treatment for TDI. The majority of the parents could not recall the history, month, and time of the injury. Parents being unaware of TDI was the main reason for not seeking treatment. The low number of children with trauma seeking dental care clearly indicates the need for their parents and teachers to be informed about the preventive measures to be taken in terms of various age groups. They should also be informed about what to do in the event of trauma and why there is a need to see a dentist immediately. This data will be useful in the planning of state- and national-level policy guidelines along with preventive measures regarding TDI.
